# Research on Rotational Angle Measurement for the Smart Wheel Force Sensor

**DOI:** 10.3390/s20041037

**Published:** 2020-02-14

**Authors:** Dong Wang, Siwei Chen, Xuanpeng Li, Weigong Zhang, Haolong Jin

**Affiliations:** 1 School of Instrument Science and Engineering, Southeast University, Nanjing 210096, China; yzchensiwei@126.com (S.C.); li_xuanpeng@seu.edu.cn (X.L.); 101001418@seu.edu.cn (W.Z.); 2 China North Vehicle Research Institute, Beijing 100072, China; hljin@noveri.com.cn

**Keywords:** wheel force sensor, rotational angle measurement, accelerometer, error analysis

## Abstract

The measurement of the rotational angle of the wheel is critical for the smart wheel force sensor (SWFS) to obtain the wheel forces defined in the vehicle coordinates. To simplify the structure of the SWFS and overcome the shortcomings of the traditional angular transducer, a new method to evaluate the rotational speed of the wheel and then calculate the rotational angle is proposed in this paper. In this method, the centripetal acceleration caused by the rotation is recorded by three accelerometers and used carefully. What’s more, the possible sources of error are classified and analyzed. Simulations and stand experiment are carried out to demonstrate the effectiveness of the proposed method.

## 1. Introduction

**Figure 1:**
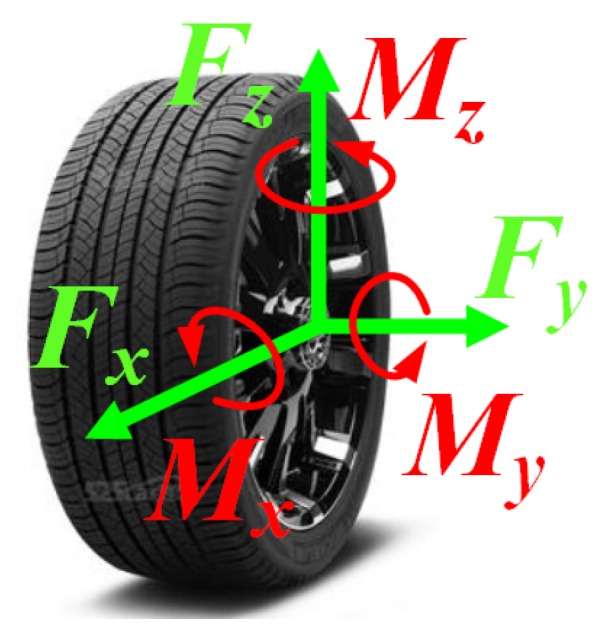
Definition of wheel forces.

The smart wheel force sensor (SWFS) is a kind of especially designed multiple dimensional force sensor used to measure the forces between the wheel and the road [1,2,3]. These wheel forces (as shown in Figure 1), including longitudinal force $F_{x}$, lateral force $F_{y}$, vertical force $F_{z}$, heeling moment $M_{x}$, twist torque $M_{y}$ and aligning torque $M_{z}$, have been proved absolutely critical for the design and testing of vehicles in the past decades [4,5]. For example, $F_{x}$ is helpful to design an antilock brake system [6,7,8], and $F_{z}$ could be used in load spectrum analysis [9,10]. Now that in-wheel motors are finding increasingly utilization in new intelligent vehicles, the need for high precision measurement and control of the wheel forces is becoming more important than ever [11,12,13].

Since the SWFS is installed on the wheel and moving with it [14,15], the direct output force of the SWFS and the real wheel force are in separate wheel coordinates and vehicle coordinates [16,17], thus the direct output force of the SWFS should be converted to the vehicle coordinates, and the rotational angle of the wheel is critical to identify the relative motion of the two coordinates. The usage of an encoder is the traditional way to measure the rotational angle, but it brings extra errors during the steering of the wheel [18,19]. The gyroscope is an alternative method, but the measuring rate (less than ± 2000°/s in common for most micro-electro-mechanical system (MEMS) gyroscopes in market) is not practical under driving wheel conditions, which are to 6000°/s when the driving speed of the vehicle is 120 km/h, so in this paper, we propose a new method to evaluate the relative rotational angle between these coordinates. Unlike the traditional gratings or Hall type angle sensors, no additional fixed part is needed for this method, thus it could support wireless data transmission in the SWFS [18].

The remainder of this paper is organized as follows: The relationship between the real wheel forces and the outputs of the SWFS will be explained in Section 2. Section 3 elaborates the details of the proposed method, which include the angle evaluation and the calibration strategy. In Section 4 the performance of this method has been analyzed and tested through numerical simulations and the stand experiments. Section 5 concludes the paper.

## 2. Coordinates Relationship in the SWFS

**Figure 2:**
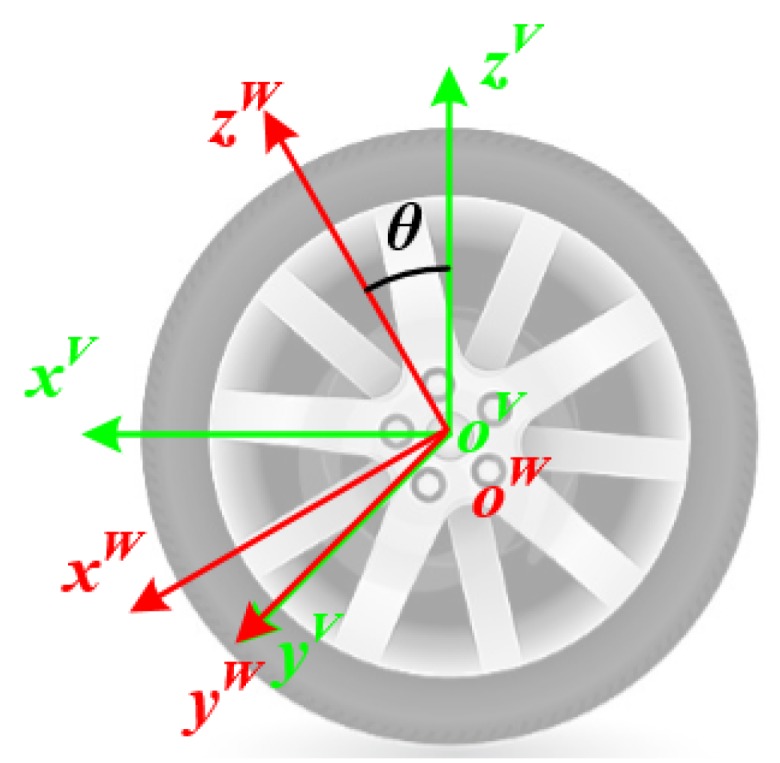
Coordinates in the SWFS.

In order to obtain the real wheel forces by the SWFS, two coordinates, called the vehicle coordinate ($o^{V}$) and the wheel coordinate ($o^{W}$), need to be defined firstly, as shown in Figure 2 [20]. In terms of $o^{V}$, it is fixed to the vehicle and the wheel forces are defined in it. Its origin is located in the center of the wheel. The axes $o^{V}x^{V}$, $o^{V}y^{V}$, and $o^{V}z^{V}$ coincide with the orientations of $F_{x}$, $F_{y}$, and $F_{z}$, which point forwards, sideways, and straight up separately, but unfortunately, as discussed above, the SWFS can only give the forces in the coordinate which is fixed to the wheel and the coordinate $o^{W}$ is then defined for this reason. The origin of $o^{W}$ is also in the rotational center of the wheel. The axis $o^{W}y^{W}$ coincides with $o^{V}y^{V}$, in other words, the rotational axis. The remaining axes ($o^{W}x^{W}$, and $o^{W}z^{W}$) are both in the rotational plane and perpendicular to each other. When the vehicle stops on the ground with a specific position, which means that the axis $o^{W}z^{W}$ happens to point up, $o^{V}$ and $o^{W}$ are equivalent. If the vehicle starts to move, the rotation of the wheel forms an angle θ to separate $o^{W}$ from $o^{V}$. Thus, the relationship between these two coordinates could be represented by Equation (1), where $C_{W}^{V}$ is the transformation matrix.

(1) $$\left[\begin{array}{c} x^{V}\\ y^{V}\\ z^{V} \end{array}\right]=C_{W}^{V}\left[\begin{array}{c} x^{W}\\ y^{W}\\ z^{W} \end{array}\right]=\left[\begin{array}{ccc} \cos\theta & 0 & \sin\theta \\ 0 & 1 & 0\\ -\sin\theta & 0 & \cos\theta \end{array}\right]\left[\begin{array}{c} x^{W}\\ y^{W}\\ z^{W} \end{array}\right]$$

(2) $$F^{V}=\left[\begin{array}{cc} C_{W}^{V} & 0\\ 0 & C_{W}^{V} \end{array}\right]F^{W}$$

In this case, we can conveniently get the real wheel forces ($F^{V}$), which are defined in $o^{V}$, from the output forces of the SWFS ($F^{W}$), which are measured in $o^{W}$, by Equation (2), where $F^{V}={\left[F_{x}\;F_{y}\;F_{z}\;M_{x}\;M_{y}\;M_{z}\right]}^{’}$ and $F^{W}={\left[F_{x}^{W}\;F_{y}^{W}\;F_{z}^{W}\;M_{x}^{W}\;M_{y}^{W}\;M_{z}^{W}\right]}^{’}$. It is worth noticing in Equations (1) and (2) that the evaluation of the rotational angle θ is the key factor in the date processing of the SWFS. What’s more, recent research shows that even the same angle error might bring considerably more error to a smaller wheel force (like $F_{x}$ the traction force) than to a larger one (like $F_{z}$ the positive pressure) [20]. This makes it more important to do a high precision rotational angle evaluation.

## 3. Rotational Angle Evaluation

The traditional way to obtain the rotational angle of the wheel is based on the encoder or rotating speed sensor. These methods require either additional fixed devices (gratings or Hall type), which causes installation errors, or costly sensors (wide range gyroscopes), which increase the total cost of the SWFS [21]. In this paper, three double-axis accelerometers are used to calculate the rotational speed of the wheel, and then the rotational angle is given by integration [22,23].

### 3.1. Rotational Speed Evaluation

**Figure 3:**
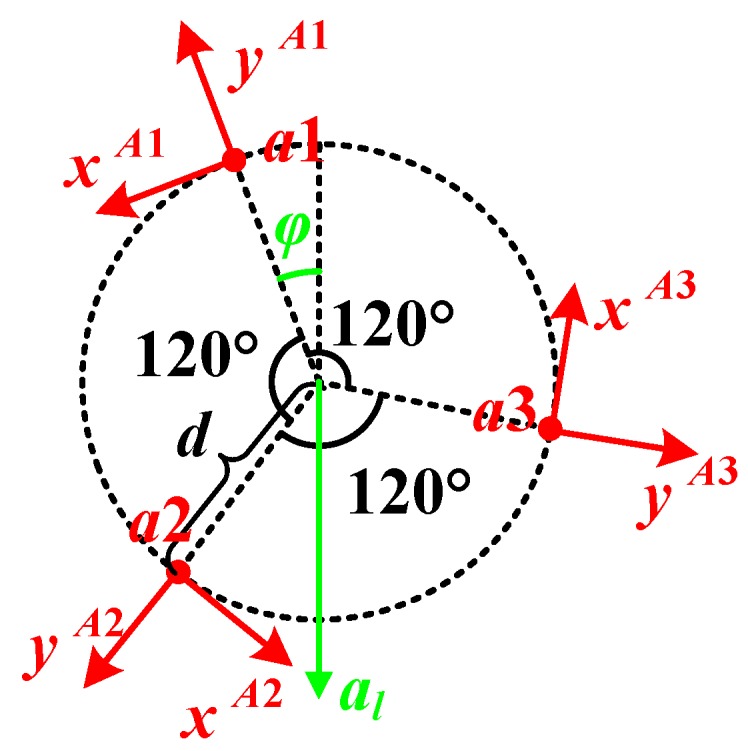
Distribution of the accelerometers.

As shown in Figure 3, accelerometers $a_{1}$, $a_{2}$ and $a_{3}$ are distributed per 120° on the circle with the radius of d, and the center of the circle locates on the rotational center of the wheel. since the sensitive axes point to the normal and the tangential respectively, only the $y^{Ai}$-axis (i=1,2,3) could sense the centripetal acceleration caused by the rotation.

In this situation, the outputs of the accelerometers on the $y^{Ai}$-axes are represented as follows:

(3) $$a_{y1}=-\omega^{2}d-a_{l}\cos\varphi$$

(4) $$a_{y2}=-\omega^{2}d-a_{l}\cos\left(\varphi +\frac{2\pi }{3}\right)=-\omega^{2}d+\frac{1}{2}a_{l}\cos\varphi +\frac{\sqrt{3}}{2}a_{l}\cos\varphi$$

(5) $$a_{y3}=-\omega^{2}d-a_{l}\cos\left(\varphi +\frac{4\pi }{3}\right)=-\omega^{2}d+\frac{1}{2}a_{l}\cos\varphi -\frac{\sqrt{3}}{2}a_{l}\cos\varphi$$

where $a_{l}$ is the projection of the linear acceleration on the rotational plane. It could be in any direction, and φ represents the angle between the prolonged line in opposite direction of $a_{l}$ and the $y^{A1}$-axis, which means the angle between $a_{l}$ and the $y^{A1}$-axis is π−φ. From Equations (3)–(5), the rotational speed of the wheel is given by Equation (6):

(6) $$\omega =\sqrt{\frac{a_{y1}+a_{y2}+a_{y3}}{-3d}}$$

### 3.2. Evaluation Error Analysis

It should be pointed out that Equation (6) is the result of a theoretical derivation. However, many factors, which could be called error sources, might bring possible errors of the rotational speed evaluation and further affect the accuracy of the rotational angle in the real use [24]. All these error sources may be classified into two types. The first one is the machining errors, which means the errors caused by the machining precision during the production processes of the SWFS, including attitude error, angular distribution error and rotational radius error. The second one is the installation errors, which means the errors caused by the use of the SWFS, including eccentric error. All these errors will be analyzed in the remainder of this section.

#### 3.2.1. Attitude Error

**Figure 4:**
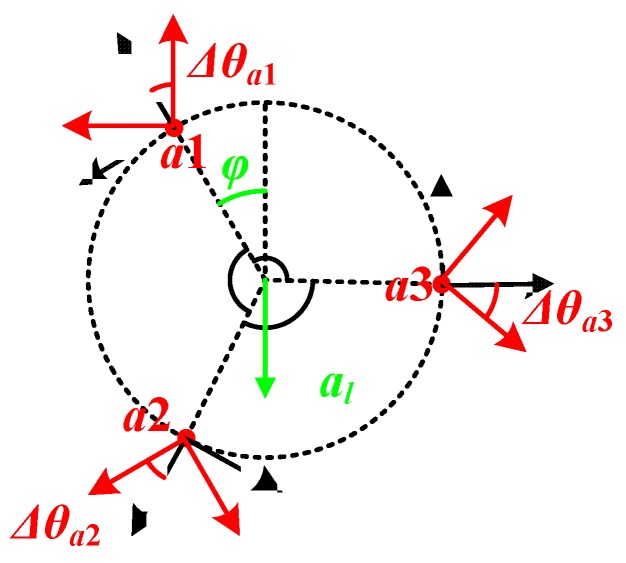
Description of the attitude error.

Due to the machining errors, the $y^{Ai}$-axis (i=1,2,3) of the accelerometers might not point to the normal strictly, and there exists the attitude error $\Delta \theta_{ai}$ (i=1,2,3) as shown in Figure 4.

In this case, the angular acceleration is divided into both sensitive axes of each accelerometer, which makes the outputs of accelerometers follow Equation (7), where i=1,2,3:

(7) $$\{\begin{array}{c} a_{xai}=-\omega^{2}d\sin\Delta \theta_{ai}+a_{l}\sin\left[\varphi -\Delta \theta_{ai}+\left(i-1\right)\frac{2\pi }{3}\right]\\ a_{yai}=-\omega^{2}d\cos\Delta \theta_{ai}-a_{l}\cos\left[\varphi -\Delta \theta_{ai}+\left(i-1\right)\frac{2\pi }{3}\right] \end{array}$$

It is obvious that when the SWFS is rotating with a specific speed, the waveforms of both $a_{xai}$ and $a_{yai}$ will appear to be like a sine wave, and the mean values of $a_{xai}$ and $a_{yai}$ are $-\omega^{2}d\sin\Delta \theta_{ai}$ and $-\omega^{2}d\cos\Delta \theta_{ai}$, respectively. Thus, we could spin the SWFS steadily and record the outputs of the accelerometers. This way, the attitude error $\Delta \theta_{ai}$ could be calibrated by Equation (8):

(8) $$\Delta \theta_{ai}=\tan^{-1}\frac{a_{xai\_mean}}{a_{yai\_mean}}=\tan^{-1}\frac{-\omega^{2}d\sin\Delta \theta_{ai}}{-\omega^{2}d\cos\Delta \theta_{ai}}$$

After the calibration of $\Delta \theta_{ai}$, the attitude error could be eliminated by coordinate transformation, as shown in Equation (9):

(9) $$\left[\begin{array}{c} a_{xi}\\ a_{yi} \end{array}\right]=\left[\begin{array}{cc} \cos\Delta \theta_{ai} & -\sin\Delta \theta_{ai}\\ \sin\Delta \theta_{ai} & \cos\Delta \theta_{ai} \end{array}\right]\left[\begin{array}{c} a_{xai}\\ a_{yai} \end{array}\right]$$

#### 3.2.2. Angular Distribution Error

**Figure 5:**
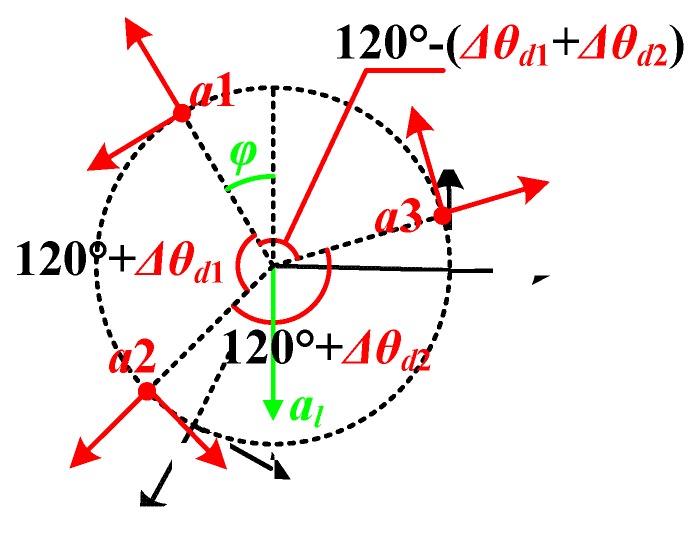
Description of the angular distribution error.

If the distribution of the three accelerometers is not equally spaced on the circle, which is called angular distribution error as shown in Figure 5, the outputs of the accelerometers on the $y^{Ai}$-axes are represented as Equations (10)–(12).

(10) $$a_{yd1}=-\omega^{2}d-a_{l}\cos\varphi$$

(11) $$a_{yd2}=-\omega^{2}d-a_{l}\cos\left(\varphi +\frac{2\pi }{3}+\Delta \theta_{d1}\right)$$

(12) $$a_{yd3}=-\omega^{2}d-a_{l}\cos\left(\varphi -\frac{2\pi }{3}+\Delta \theta_{d1}+\Delta \theta_{d2}\right)$$

where $\Delta \theta_{d1}$ and $\Delta \theta_{d2}$ are the angular errors between the accelerometers, and Equation (6) will become Equation (13):

(13) $$\omega =\sqrt{\frac{a_{yd1}+a_{yd2}+a_{yd3}+a_{l}\left[\cos\varphi +\cos\left(\varphi +\frac{2\pi }{3}+\Delta \theta_{d1}\right)+\cos\left(\varphi -\frac{2\pi }{3}+\Delta \theta_{d1}+\Delta \theta_{d2}\right)\right]}{-3d}}$$

When the angular errors are small enough, Equation (13) could be simplified into Equation (14), where $\sin\Delta \theta_{di}$ is replaced by $\Delta \theta_{di}$, and $\cos\Delta \theta_{di}$ is replaced by 1 (i=1,2):

(14) $$\omega \approx \sqrt{\frac{a_{yd1}+a_{yd2}+a_{yd3}-a_{l}\left[\sin\left(\varphi +\frac{2\pi }{3}\right)\Delta \theta_{d1}+\sin\left(\varphi -\frac{2\pi }{3}\right)\left(\Delta \theta_{d1}+\Delta \theta_{d2}\right)\right]}{-3d}}$$

It is clear that if Equation (6) is still used in this situation, the evaluation error of the rotational speed $\omega_{ed}$ would be as follows:

(15) $$\omega_{ed}=\omega_{d}-\omega =\sqrt{\frac{a_{yd1}+a_{yd2}+a_{yd3}}{-3d}}-\sqrt{\frac{a_{yd1}+a_{yd2}+a_{yd3}-f_{d}\left(\varphi \right)}{-3d}}$$

where $f_{d}\left(\varphi \right)$ represents $\sin\left(\varphi +\frac{2\pi }{3}\right)\Delta \theta_{d1}+\sin\left(\varphi -\frac{2\pi }{3}\right)\left(\Delta \theta_{d1}+\Delta \theta_{d2}\right)$. Equation (15) shows that the evaluation error changes with angle φ even when the $\Delta \theta_{di}$ (i=1,2) is constant. By calculating the derivative of $f_{d}\left(\varphi \right)$, the maximum of $\omega_{ed}$ appears when $f_{d}\left(\varphi \right)$ reaches its minimum, that is, φ equals to $\tan^{-1}\frac{2\Delta \theta_{d1}+\Delta \theta_{d2}}{\sqrt{3}\Delta \theta_{d2}}$.

**Figure 6:**
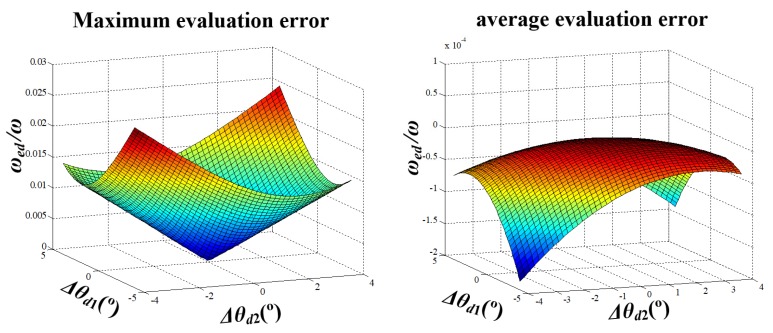
The evaluation error of the rotational speed with angular changing distribution error.

**Figure 7:**
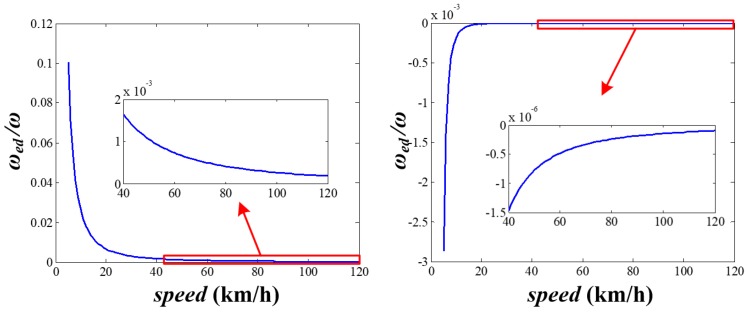
The evaluation error of the rotational speed with changing vehicle speed.

The maximum and average evaluation error of the rotational speed is as shown in Figure 6, with d=0.1 m, ω=500°/s (corresponding to 10 km/h for a normal car). It can be seen in this figure that a larger angular distribution error causes a larger evaluation error. For example, when $\Delta \theta_{di}=-\;4{}^{\circ}$ (i=1,2), the maximum and average evaluation errors are 2.6% and −0.0187%, respectively. Furthermore, Figure 7 gives the evaluation error with the changing vehicle speed. By comprehensive consideration of Figure 6 and Figure 7, if the SWFS is rotating with the wheel normally, the average evaluation error of the rotational speed caused by angular distribution error is quite small except for some specific φ and when vehicle speed is above 40 km/s, both the maximum and average evaluation error are acceptable for the usage of the SWFS (less than 2‰). That means the SWFS might have higher precision with a higher speed.

#### 3.2.3. Rotational Radius Error

**Figure 8:**
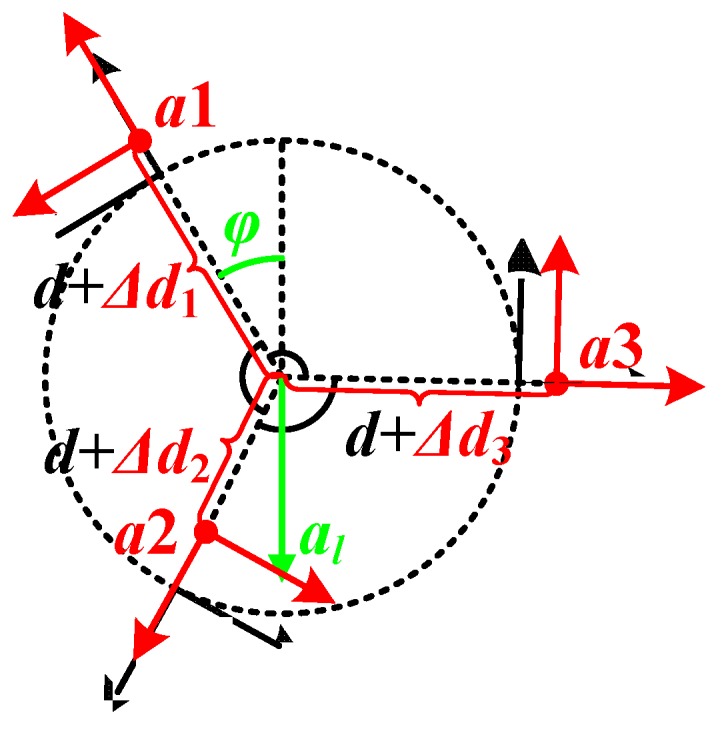
Description of the rotational radius error.

Besides the attitude and angular distribution errors, the distance from the location point of the accelerometer to the rotating center might not be strictly equal to d, as shown in Figure 8, and that brings a rotational radius error.

Equations (16)–(18) give the outputs of the accelerometers in this way:

(16) $$a_{yr1}=-\omega^{2}\left(d+\Delta d_{1}\right)-a_{l}\cos\varphi$$

(17) $$a_{yr2}=-\omega^{2}\left(d+\Delta d_{2}\right)-a_{l}\cos\left(\varphi +\frac{2\pi }{3}\right)$$

(18) $$a_{yr3}=-\omega^{2}\left(d+\Delta d_{3}\right)-a_{l}\cos\left(\varphi -\frac{2\pi }{3}\right)$$

where $\Delta d_{i}$ (i=1,2,3) is the distance error of each accelerometer, and with this error, Equation (6) will turn to Equation (19). Thus, the evaluation error of the rotational speed $\omega_{er}$ is given in Equation (20), where $\Delta D=\Delta d_{1}+\Delta d_{2}+\Delta d_{3}$. When ΔD is quite small, the linear approximation of $\omega_{er}$ is shown in Equation (21):

(19) $$\omega =\sqrt{\frac{a_{yr1}+a_{yr2}+a_{yr3}}{-3d-\left(\Delta d_{1}+\Delta d_{2}+\Delta d_{3}\right)}}$$

(20) $$\omega_{er}\left(\Delta D\right)=\omega_{r}-\omega =\sqrt{\frac{-3\omega^{2}d-\omega^{2}\left(\Delta D\right)}{-3d}}-\omega$$

(21) $$\omega_{er}\left(\Delta D\right)\approx \omega_{er}\left(0\right)+\omega_{er}^{’}\left(0\right)\Delta D=\frac{\omega }{3d}\Delta D$$

**Figure 9:**
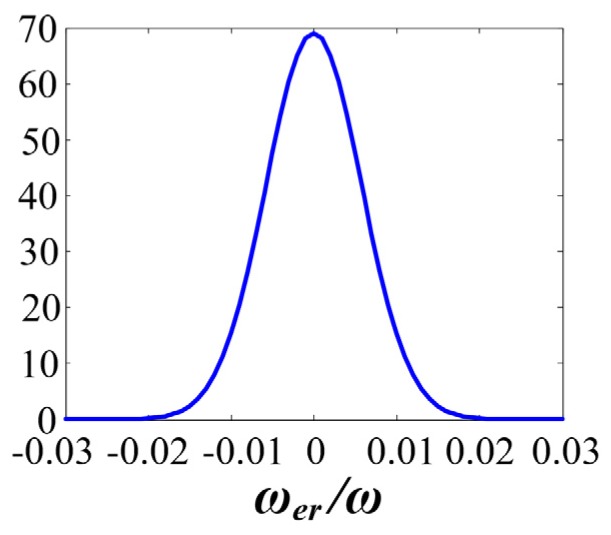
Probability density of the relative error caused by the rotational radius error.

Let $\Delta d_{i}$ (i=1,2,3) obeys normal distribution, and $P\left\{-0.002<\Delta d_{i}<0.002\right\}=95.44\%$. Then $\Delta d_{i}\sim N\left(0,0.001^{2}\right)$, and $\Delta D\sim N\left(0,0.0017^{2}\right)$. The probability density of $\frac{\omega_{er}}{\omega }*100$ is shown in Figure 9 (with d=0.1 m). This figure shows the relative error caused by rotational radius error will not extend ± 2%, with the probability more than 95%.

#### 3.2.4. Eccentric Error

**Figure 10:**
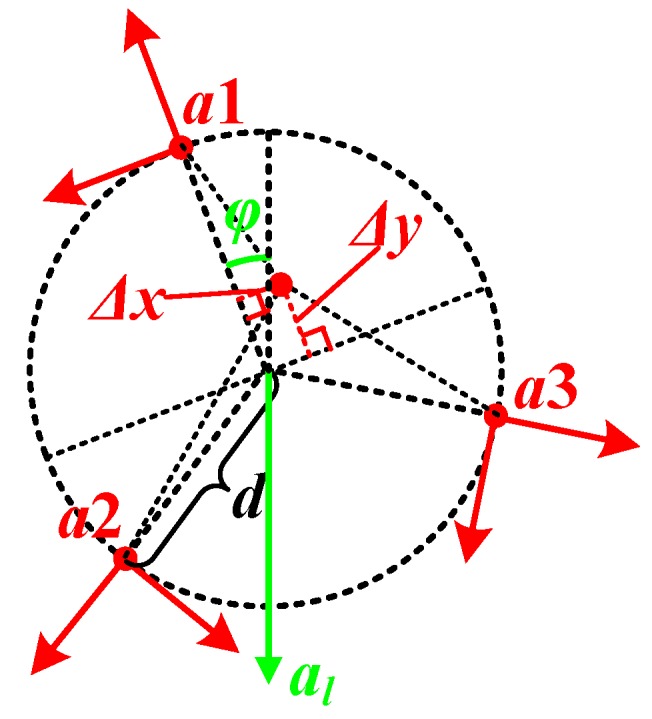
Description of the eccentric error.

In the installation of the SWFS, the real rotational center of the wheel may deviate from center of the circle which depends on the location of the three accelerometers, as shown in Figure 10. Unlike the machining error, the error caused by the installation could not be tested or calibrated in the lab, and will affect the calculated rotational speed directly, so we analyse this error separately.

In Figure 10, the deviation values are described by Δx and Δy. In this case, the outputs of the accelerometers follow Equations (22)–(24). Thus, the rotational speed could be still calculated by the form of Equation (6), as shown in Equation (25). That is to say, the eccentric error will not influence the evaluation of rotational speed. This is in accordance with the phenomena that the translation of the rotation axis does not change the rotational speed of any point on the rigid body:

(22) $$a_{ye1}=-\omega^{2}\left(d-\Delta y\right)-a_{l}\cos\varphi$$

(23) $$a_{ye2}=-\omega^{2}\left(d-\frac{\sqrt{3}}{2}\Delta x+\frac{1}{2}\Delta y\right)-a_{l}\cos\left(\varphi +\frac{2\pi }{3}\right)$$

(24) $$a_{ye3}=-\omega^{2}\left(d+\frac{\sqrt{3}}{2}\Delta x+\frac{1}{2}\Delta y\right)-a_{l}\cos\left(\varphi -\frac{2\pi }{3}\right)$$

(25) $$\omega =\sqrt{\frac{a_{ye1}+a_{ye2}+a_{ye3}}{-3d}}$$

## 4. Simulations and Tests

**Figure 11:**
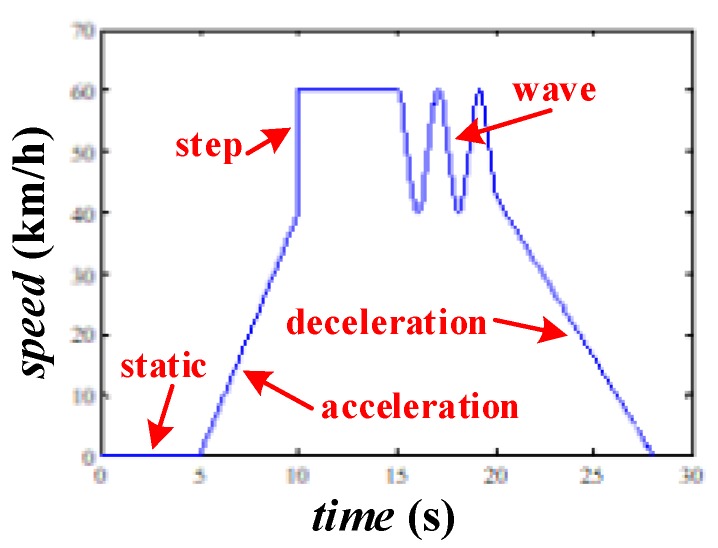
Vehicle speed curve.

Numerical simulations are carried out in this section to examine the performance of the proposed rotational angle measurement method. First of all, the designed vehicle speed curve shown in Figure 11 is divided into five segments, including static, uniform acceleration, step, wave and deceleration, to test the method more fully. In all simulations, the location error of the accelerometer ($\Delta p_{e}$) obeys normal distribution and is within ± 2 mm for the probability more than 95% ($\Delta p_{e}\sim N\left(0,0.001^{2}\right)$. Thus, the rotational radius error, which depends on $\Delta p_{e}$ directly, satisfies $\Delta d_{i}\sim N\left(0,0.001^{2}\right)$ (i=1,2,3). The angular distribution error, which depends on the equation $d*\Delta \theta_{di}=\Delta p_{e}$ (i=1,2), satisfies $\Delta \theta_{di}\sim N\left(0,{\left(\frac{0.001}{d}\right)}^{2}\right)$ (i=1,2). Also, the diameter of the wheel is set as 0.7 m to match the size of an R16 tire.

**Figure 12:**
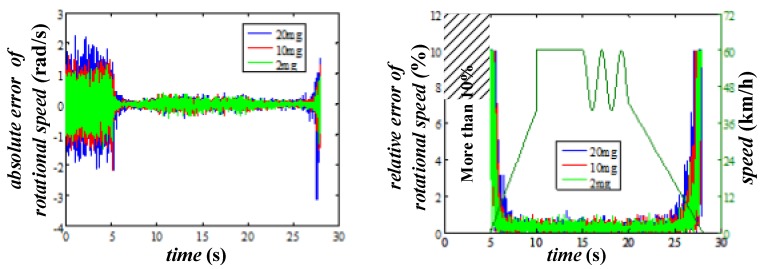
The evaluation error of rotational speed with different output noise.

In this case, the effects caused by different output noise of the accelerometer are tested (with d=0.1 m), and the absolute and relative errors are shown in Figure 12. Two facts can be deduced from in this figure. The first one is the output noise of the accelerometer has little effect on the accuracy of the rotational speed evaluation, especially when the speed exceeds 15 km/h, that means there is no need to pay for the expensive high accuracy accelerometers. The second one is the evaluation accuracy of the proposed method is acceptable at high speed, but is almost useless if the vehicle speed is less than 10 km/h.

**Figure 13:**
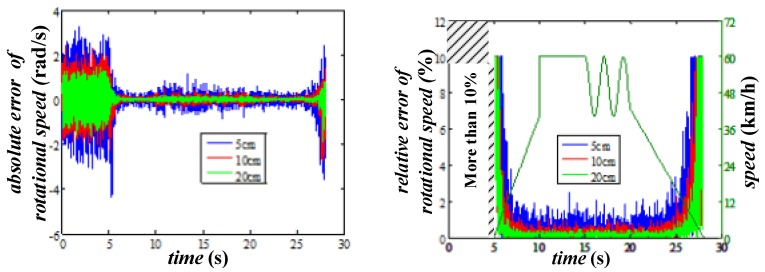
The evaluation error of rotational speed with different rotational radii.

The results of the simulations with different rotational radii and fixed output noise (20 mg) are shown in Figure 13. It could be noted that the larger rotational radius makes the smaller evaluation error, which means the circle where the accelerometers locate should be as large as possible within acceptable limit of the size of the wheel. Besides, the results have proved again that if we want to use this method in practical, another way must be found to deal with the low speed situation.

**Figure 14:**
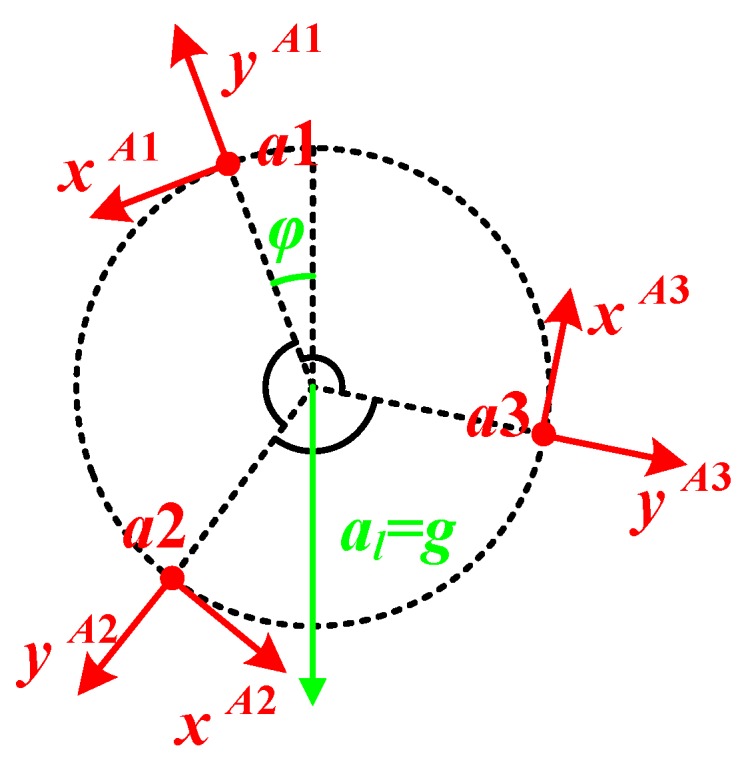
Rotational angle evaluation in low speed.

When the vehicle is static or driving slowly (not exceeding 15 km/h), the acceleration acting on the wheel could be considered as gravity only. Thus, the output of the accelerometer $a_{1}$ on $x^{A1}$-axis could be used to calculate the rotational angle of the wheel directly, as shown in Figure 14. The rotational angle φ equals $\sin^{-1}\left(\frac{a_{x1}}{g}\right)$.

Except for the low speed scenario, another problem that must be solved is that the bias of the accelerometer may bring an accumulated error in the calculation of the rotational angle over time by the integration of the rotational speed. A practical approach is to correct the evaluated rotational angle each rotation by 2π with the help of the output of a magnetometer, since the magnetic field changes periodically with the rotation of the wheel.

**Figure 15:**
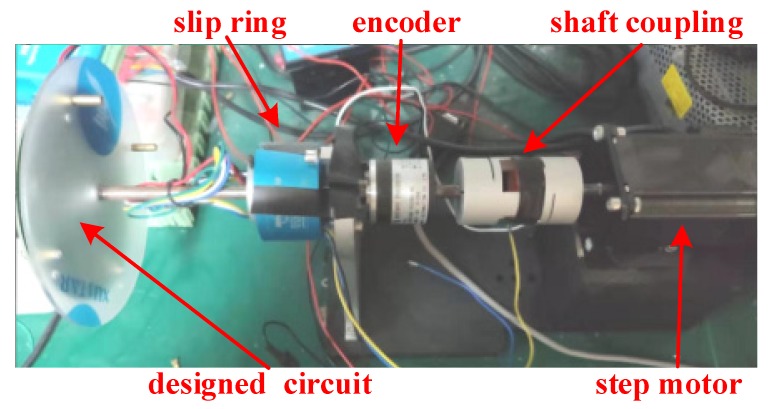
The designed test stand.

For further analysis of the performance of the proposed method atest stand is designed as shown in Figure 15. In this stand, the accelerometers and the magnetometer are installed and distributed on the circuit as mentioned above. The accelerometer we choose here is an adxl357, which has high accuracy (80$\;\mu g/\sqrt{\mathrm{Hz}}$) and an adjustable range (± 10 g, ± 20 g and ± 40 g (selected)). The radius is set as 3 cm, and a 16 bit ADC is used to record the outputs of the accelerometers. In this case, the measuring range of the proposed method is ± 6532°/s with a sensitivity of 0.2°/s, which could totally satisfy the needs of the wheel rotational speed measurements. The circuit is rotated by a step motor. In addition, a slip ring is used to transfer the power and the signal, and output of the encoder is considered as the ground truth to compare with the evaluated rotational angle.

**Figure 16:**
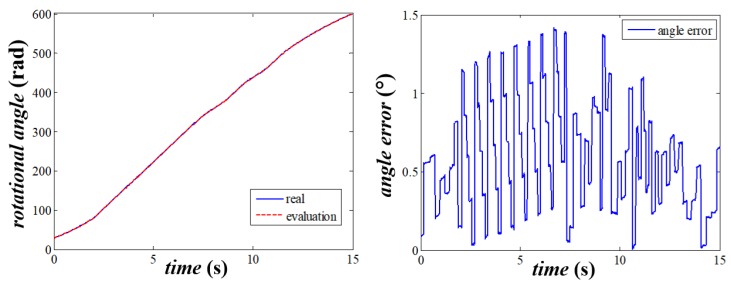
The results of the stand experiments.

The results of the stand experiment are shown in Figure 16. Tanks to the periodic correction, the absolute error of the evaluated rotational angle is within 1.5°, which satisfies the needs of the usage of the SWFS.

## 5. Conclusions

In this paper, a new method to evaluate the rotational angle of a wheel for use in the SWFS is proposed. Firstly, three accelerometers are distributed reasonably in the rotational plane of the wheel. Secondly, the outputs of the accelerometers which reflect the centripetal acceleration are used to evaluate the rotational speed. Then the rotational angle of the wheel can be obtained by the integration. Moreover, the rotational angle errors caused by slow speed and the bias of the accelerometer are modified by the acceleration of gravity and magnetometer. The simulation and the stand experiment show that the new method performs well in the real use. In this paper we sought a balance between accuracy and cost. There are two points which deserve special mention for this. The first one concerns the number of accelerometers. Any number of the accelerometers (at least two) distributed radially on a circle could provide the rotational angle calculation with a method similar to the proposed one. The more accelerometers used, the more accurate the results obtained could be, since the random errors, like the output error of the accelerometer and the rotational radius error, will be eliminated. The second one is about the calculation error. Although the sources of error are analyzed, they could not be calibrated and eliminated until now, except for the attitude error, which limits the accuracy of the rotational angle. This problem will be tackled as a crucial issue in our future work.
